# Asymptomatic uterine perforation and IUD migration to the broad ligament: A case report

**DOI:** 10.1097/MD.0000000000033857

**Published:** 2024-02-16

**Authors:** Guifeng Jia, Xiaojing Liu, Yanling He, Peng Du, Zhiwei Sun, Wei Chai

**Affiliations:** a Department of Gynecology and Obstetrics, The First Hospital of Jilin University, Changchun, China.

**Keywords:** case report, intrauterine contraceptive device, migration, uterine perforation

## Abstract

**Rationale:**

Uterine perforation is a serious complication of intrauterine contraceptive device (IUD) placement. However, as complete uterine perforation and extrauterine migration may remain asymptomatic, thorough localization of the IUD is important prior to reinsertion.

**Patient concerns:**

A 33-year-old patient who has had 4 IUD insertions, wherein the location of the first IUD (inserted 14 years ago) was not identified prior to reinsertion and replacement of the subsequent three. She presented to hospital with a 6-month history of abdominal pain. Pelvic ultrasonography (US), radiography, hysteroscopy and laparoscopy examinations confirmed that a retained migrated IUD in the right broad ligament.

**Diagnosis:**

Uterine perforation, IUD migration to the broad ligament.

**Interventions:**

The patient underwent hysteroscopy and laparoscopy.

**Outcomes:**

Both IUDs were successfully removed without any complications.

## 1. Introduction

The intrauterine device (IUD) is a long-term contraceptive tool, which is safe, effective, economical, rapidly reversible, and technically simple to insert. It does not interfere with sexual intercourse, and has no impact on future pregnancies following removal. Such properties render it the most widely used and accepted method of contraception among women of childbearing age.

Displacement is a serious complication of IUDs. This may range from partial or complete embedment into the uterine myometrium, to migration into the abdominal or pelvic cavity.^[1]^ There are many causes of IUD displacement, such as unfavorable uterine conditions in terms of size, position, and physiological characteristics, as well as the presence of scar tissues or poor myometrium healing post-surgery. Furthermore, uterine shrinkage can increase the risk of uterine perforation. Complications of IUD displacement can include difficulty in removal, and in severe cases, uterine perforation, damage of adjacent organs, hemorrhage, and infection, which can negatively impact the physical and mental wellbeing of patients.

We hereby report a patient who presented with a normal IUD in situ, and a retained migrated IUD in the pelvic cavity.

## 2. Case report

A 33-year-old patient was referred to our department following a 6-month history of intermittent lower abdominal pain, with findings suggestive of a migrated IUD 2 weeks ago on abdominal X-ray at a local hospital.

In terms of gynecological history, the patient reported menarche at 14 years of age, with cycles lasting 24 to 25 days, and menstrual periods lasting 2 to 5 days. She denied any dysmenorrhea, and her last menstrual period was July 01, 2020. Her first child was born by natural delivery 14 years ago, and her first IUD was inserted during the lactation period. She underwent artificial abortion 1 year later due to an unintended pregnancy; however, as the IUD was not detected during the operation, it was considered lost without further investigations. Since the abortion, a second IUD was reinserted, and was subsequently replaced twice.

**Figure 1.**
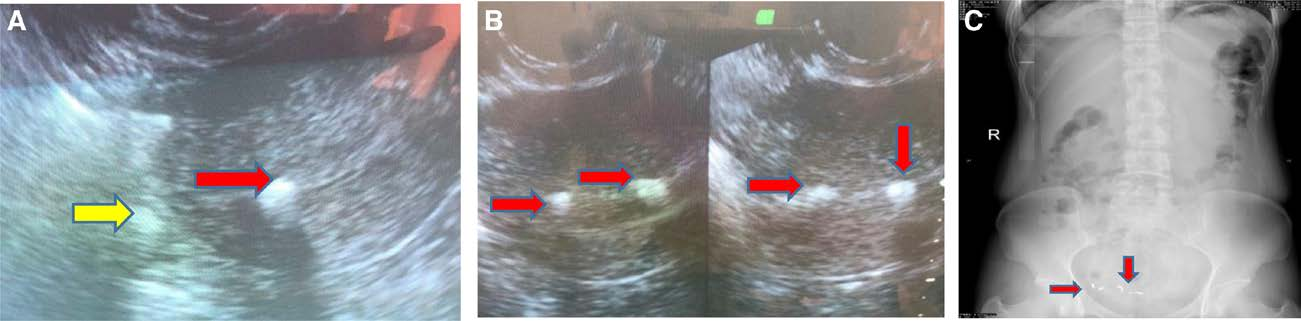
(A) Metal-like hyperechoic spots within (red arrow) and outside of (yellow arrow) the myometrium of the right side of the uterus on ultrasonography. (B) An IUD normally positioned in uterine cavity (red arrow) on ultrasonography. (C) Two irregular high-density shadows in the pelvic cavity (red arrow) on abdominal X-ray.

Transvaginal color ultrasound demonstrated two metal-like hyperechoic spots, one within, and the other outside of, the myometrium of the right uterus (Fig. 1A). A second IUD was found correctly positioned in the uterine cavity (Fig. 1B). Abdominal X-ray verified the presence of 2 irregular high-density shadows in the pelvic cavity (Fig. 1C). Based on such findings, a migrated IUD was suspected.

**Figure 2.**
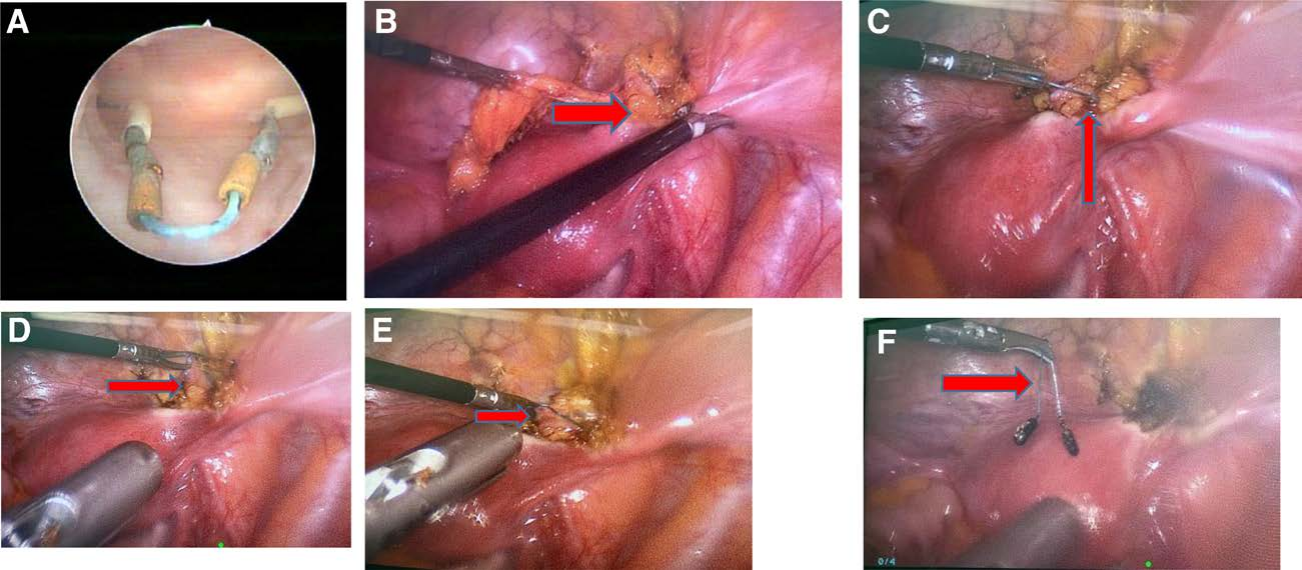
(A) The normal V-shaped IUD in the uterine cavity on hysteroscopy. (B) Adhesions of the separated greater omentum with the anterior lobe of the right round ligament and the right broad ligament (red arrow) on laparoscopy. (C) One arm of the migrated IUD in the right round ligament (red arrow). (D) The other arm of the migrated IUD in the serous layer of the right anterior wall of the uterus (red arrow). (E) The tail of the migrated IUD in the right broad ligament (red arrow). (F) Complete removal of the migrated IUD (red arrow).

Combined hysteroscopy and laparoscopy were therefore performed under general anesthesia. Hysteroscopy confirmed the presence of a normal V-shaped IUD within the uterine cavity (Fig. 2A). Laparoscopy demonstrated adhesions of the greater omentum with the right round ligament, the right anterior lobe of the broad ligament, and the right anterior abdominal wall (Fig. 2B). Separation of the adhesions exposed the migrated IUD, with the arms attached to the right round ligament and the serous layer of the right anterior wall of the uterus, respectively, while the tail to the right broad ligament (Fig. 2C–E). A diagnosis of a retained migrated IUD in the broad ligament was therefore confirmed. The migrated IUD was successfully removed with no injuries made to the adjacent organs (Fig. 2F). The patient recovered without any complications.

## 3. Discussion

IUD is a widely used method of contraception, with an estimated number of 150 million users worldwide particularly in developing countries.^[2]^ While it is generally considered safe, serious complications such as IUD displacement can occur. Potential consequences may include partial or complete embedment into the uterine myometrium, and uterine perforation resulting in extrauterine migration. Reported extrauterine migration sites have included the omentum, broad ligament, rectosigmoid, peritoneum, bladder, and even the inguinal region.^[3]^ The reported incidence of uterine perforation ranges between 0.2 to 3.6 per 1000 inserts^[4–6]^; however, as some uterine perforations may remain asymptomatic, the actual incidence may be higher.^[7]^ Migrated IUDs are often an incidental finding, but may manifest as vague symptoms such as lower abdominal pain, lumbosacral pain, and urinary symptoms.^[8]^

Uterine perforation may be associated with a multitude of factors, including the timing of IUD insertion, IUD type, skills of the attending physician, anatomy of the uterus and cervix, postpartum status, and history of uterine perforation.^[9,10]^ Importantly, lactation and abortion, which were both present in our patient, have been identified as risk factors for uterine perforation with IUD insertion. As per the case report by Patel et al^[11]^ on a 21-year-old patient with immediate IUD placement following spontaneous labor, uterine perforation occurred 18 months later, with findings of an inflammatory mass involving the right iliac vessel, ileus, and right ureter. The IUD was removed by laparotomy. As such, a 4-week interval post-delivery has been recommended as a cutoff for safe IUD placement.^[12]^

Several IUD types are used in China, including T-shaped, V-shaped, O-shaped, palace-shaped, and flower-shaped IUDs. Insertion of different IUD types involve different techniques. A V-shaped IUD was used in our patient, which is high in elasticity, with relatively thick copper arms consisting of copper particles. Excessive pressure during placement may result in direct compression of the arms onto the uterine myometrium, increasing the long-term risk of incarceration and perforation.^[8]^ Unfavorable uterine conditions following cesarean section, including the presence of scar tissues in the uterus, excessive uterine flexion, uterine malformation, cervical stenosis may also lead to a predisposition of uterine damage during IUD placement, increasing the risk of uterine perforation and IUD migration. The inadequate assessment of size and position of the uterus in relative to the IUD inserted may be a contributing factor as well.

The medical history should mainly include the birth history, IUD type, timing of IUD placement, complications during IUD insertion, frequency of follow-ups following IUD placement, and IUD-related side effects such as uterine shedding and displacement. The development of severe abdominal pain, gross hematuria, unintended pregnancies, or recurrent lower abdominal pain, urinary tract infections, and abnormal vaginal bleeding should raise the suspicion of IUD deviation from normal position. Ultrasound is considered first-line for the assessment of IUD migration. It can determine whether the IUD has been expulsed, displaced, or embedded. In the case of extrauterine migrations, other imaging modalities including X-ray, computed tomography, and magnetic resonance imaging would be indicated. In our patient, the migrated IUD was identified by ultrasonography and adjunctive abdominal X-ray.

The need for the removal of migrated IUDs remains controversial. The World Health Organization advocates for the removal of all extrauterine IUDs, regardless of its type and location.^[13]^ This is supported by Kho and Chamsy and Balci et al^[5,14]^ On the contrary, Markovitch and Kaislasuo et al^[15,16]^ proposed that surgical interventions are not indicated for asymptomatic patients. Our study corroborates with the former notion that all migrated IUDs should be immediately removed regardless of symptoms. This is due to the risk of adhesions, which can result in dysmenorrhea, ectopic pregnancies, chronic pelvic pain, infertility, as well as intra-abdominal complications such as intestinal obstruction or perforation, and bladder injuries.

Migrated IUDs may be removed via the laparoscopic or laparotomic approach. Laparoscopic surgery has the advantages of minimal invasiveness and shorter hospital stays. However, laparotomy may be indicated in the case of severe adhesions, perforation of adjacent organs, or failure of IUD removal via laparoscopy.

IUD represents the main contraceptive measure for women of childbearing age. Albeit a minor procedure, it can associate with the risk of migration, which can be detrimental to the physical and mental wellbeing of patients. Our study suggests that immediate IUD placement during lactation and post-abortion should be avoided. The decision for IUD insertion, and the procedure itself, should also be done by well-trained professionals. Regular follow-ups post-procedure are essential for early detection of migrated IUDs. Upon suspicion of IUD displacement, thorough history and physical examination are essential, and should be followed by imaging for accurate localization of the IUD. We further propose that all migrated IUDs should be surgically removed regardless of clinical symptoms.

## Acknowledgments

We would like to thank the native English speaking scientists of Elixigen Company (Huntington Beach, California) for editing our manuscript.

## Author contributions

**Investigation:** Xiaojing Liu.

**Validation:** Yanling He, Peng Du, Zhiwei Sun.

**Writing – original draft:** Guifeng Jia.

**Writing – review & editing:** Wei Chai.
